# The Safety and Effectiveness of Infliximab Biosimilar in Managing Rheumatoid Arthritis: A Real-Life Experience from Jordan

**DOI:** 10.1155/2022/3406783

**Published:** 2022-08-26

**Authors:** Khaldoon Alawneh, Abdel-Hameed Al-Mistarehi, Ali Qandeel, Ruba Jaber, Safwan Alomari, Khalid A. Kheirallah

**Affiliations:** ^1^Department of Internal Medicine, Faculty of Medicine, Jordan University of Science and Technology, Irbid, Jordan; ^2^Department of Public Health and Family Medicine, Faculty of Medicine, Jordan University of Science and Technology, Irbid, Jordan; ^3^Department of Rheumatology, Prince Hamzah Hospital, Amman, Jordan; ^4^Clinical Research and Development Department, Hikma Pharmaceuticals LLC, Amman, Jordan; ^5^Johns Hopkins University School of Medicine, Baltimore, Maryland, USA

## Abstract

**Background:**

Infliximab (IFX) biosimilar was the first biosimilar approved in Jordan in 2014, with limited evidence of its safety and effectiveness from the Middle East and North Africa (MENA) region. Thus, this study aimed to evaluate the safety and effectiveness of IFX biosimilar in active rheumatoid arthritis (RA) patients over 34 weeks by investigating (1) the adverse events (AEs), serious adverse events (SAEs), and therapy discontinuation and (2) the score changes of the 28-Joint Disease Activity Score (DAS28) and the Health Assessment Questionnaire Disability Index (HAQ-DI).

**Methods:**

This multicenter prospective cohort study collected clinical parameters within hospital settings every four weeks. The numbers and percentages of observed AEs and SAEs were informed. The DAS28 utilizing Erythrocyte Sedimentation Rate (ESR), HAQ-DI, and ESR were reported at baseline and 14th and 30th weeks; thus, they were reported as means (SD).

**Results:**

A total of 22 RA patients were enrolled and initiated IFX biosimilar, of which nine (41.0%) discontinued the study, but their data were analyzed up to the point of withdrawal. A total of 35 AEs were reported in 14 patients, including two (5.7%) SAEs. None of the participants discontinued treatment due to AEs. The mean (SD) score of DAS28-ESR significantly decreased from 6.55 (1.16) at baseline to 4.59 (1.45) at week 14 (*p* < 0.0001) and to 4.77 (1.09) at week 30 (*p* < 0.0001). Similarly, the mean (SD) HAQ-DI score significantly decreased from 0.95 (0.74) at baseline to 0.48 (0.62) at week 14 (*p*=0.008) and to 0.71 (0.78) at week 30 (*p*=0.483). The mean (SD) value of ESR decreased from 58.75 (26.94) at baseline to 47.92 (33.89) at week 14 (*p*=0.082) and to 39.83 (17.38) at week 30 (*p*=0.005).

**Conclusion:**

IFX biosimilar demonstrated safety and effectiveness in managing RA patients bringing real-world clinical support for biosimilars' role in rheumatology.

## 1. Introduction

An evolving number of biological Disease-Modifying Antirheumatic Drugs (DMARDs) are used to manage rheumatoid arthritis (RA). Biological agents were the hope to improve treatment targets as low disease activity in RA. However, the high cost of these drugs still limits their widespread use and thus leads to unjust access to optimal care across various regions and countries. Besides the benefit, safety, and preferences, the cost aspect has been included in the 2019 updated recommendations of the European League against Rheumatism (EULAR) for managing rheumatoid arthritis [1]. Another fundamental principle when using biological agents is that treatment decisions are usually based on disease activity, patient safety profile, and other factors such as comorbidities and progression of structural damage [1]. In many countries, the sufficiently low prices of approved biosimilar DMARDs, such as Infliximab (IFX), Adalimumab, and Etanercept, have considerably reduced the net costs of biological treatment of RA and consequently reduced the healthcare budget [1]. This has encouraged payers in these countries to reinforce the use of biosimilar based on expert opinion and experience-based guidance [1].

In developed countries, the effectiveness and safety profiles of many biologic agents, including IFX, have been extensively studied and well-established in RA patients [2–5]. However, the evidence is scarce from developing countries contributing to the limited use of biosimilars by rheumatologists. Thus, there is a need for more clinical data, particularly in real-world settings, to demonstrate the safety profile and effectiveness of biosimilars.

Jordan is one of the developing countries in the Middle East and North Africa (MENA), where access to biological medications is challenging due to their high cost [6]. Since 2014, the Jordan Food and Drug Administration (JFDA) has authorized biosimilar products with a safe, effective, and good quality profile. This is essential for autoimmune diseases such as rheumatoid arthritis, with a high severity rate and a low remission rate in Jordan [7]. Introducing safe and effective biosimilars might help reduce the costs of biological therapies in Jordan, thereby potentially reducing the treatment delay. Also, this could provide an opportunity to increase the number of rheumatology patients who have access to effective biologic medicines at a lower cost [8].

IFX biosimilar is the world's first biosimilar monoclonal antibody developed by Celltrion in Korea. It was approved by international drug regulatory agencies across about 110 countries, including the European Medicines Agency (EMA) and the US FDA [9]. The 2013 European League against Rheumatism (EULAR) indicated that IFX biosimilar has similar effectiveness and safety profiles in comparison to standard IFX original (Janssen Biotech, USA) and placed it alongside other Tumor Necrosis Factor (TNF) inhibitors in the therapeutic cascade [10]. The types and incidence rates of adverse drug reactions (ADRs) observed with IFX biosimilar and IFX original were generally similar; thus, no new safety concerns were identified [11].

JFDA approved the IFX in the treatment protocol of RA patients in August 2002, while IFX biosimilar (Manufacturer: Celltrion, Marketing Authorization Holder: Hikma Pharmaceuticals LLC) was approved in October 2014 [12]. This postauthorization study aims to evaluate the safety of IFX biosimilar in RA patients under real-world clinical practice in a developing country as part of the IFX biosimilar Risk Management Plan (RMP) for JFDA. Also, we aimed to assess the potential effectiveness of IFX biosimilar in improving the RA disease activity, inflammatory markers, and functional ability of RA patients. Our results are expected to provide a piece of evidence supporting the cost-effective decisions for IFX biosimilar medical practice in the MENA region. Also, this study could reduce the knowledge gap on the safety and effectiveness of IFX biosimilar within developing country settings.

## 2. Materials and Methods

### 2.1. Study Design

This postauthorization study has a multicenter, prospective, follow-up cohort design that evaluates the safety and effectiveness of IFX biosimilar for RA treatment in biologic naïve patients who did not receive previous biological therapy. They were administered per routine clinical practice in Jordan. This study was conducted from March 21, 2016, to April 24, 2019, and the recruitment period was from January 2017 to September 2018 in two centers in Jordan: King Abdullah University Hospital (KAUH) and Prince Hamzah Hospital (PHH). The overall study duration was 20.5 months, including 12 months (48 weeks) of the recruitment process and 8.5 months (34 weeks) of the treatment period. The total number of expected clinic visits was 11 for each patient.

After the screening period, adult patients diagnosed with active RA and biologic naïve were recruited to receive IFX biosimilar following the approved Summary of Product Characteristics (SmPC). IFX biosimilar was available and stored in the outpatient pharmacies of the participating hospitals and was administered to study patients as per routine clinical practice. To avoid missing therapy during the follow-up monitoring period, the required doses for every enrolled patient had been booked and kept in the registered stock since the patient enrollment. Thus, the treatment course was uninterrupted.

All steps related to the selection, enrollment, and treatment of these patients were in accordance with the standard medical care and the current SmPC of IFX biosimilar. The IFX biosimilar was started at the baseline with a starting dose of 3 mg/kg as an intravenous infusion, followed by additional 3 mg/kg infusion doses at 2 and 6 weeks after the first infusion, then every eight weeks up to and including week 34. No extra visits or interventions additional to the routine practice were performed. The laboratory tests were conducted at the study centers. No significant changes were conducted to the regular treatments of the enrolled patients during IFX biosimilar treatment.

### 2.2. Study Population and Ethical Approval

Patients aged ≥18 years with a clinical diagnosis of active RA as defined by the 2010 revised American College of Rheumatology (ACR) and EULAR classification criteria [13] who were biologically naïve, as they did not receive previous biological therapy, were eligible to participate in this study. At screening, eligible RA patients were required to have six or more swollen and tender joints and an Erythrocyte Sedimentation Rate (ESR) of ≥28 mm/h. Also, the enrolled patients should have been treated with a stable dose of oral methotrexate for at least 12 weeks before study enrollment and continued the dose throughout the study period with no changes. A low dose of oral prednisolone (5 to 10 mg daily) was given as a bridging therapy to DMARDs as part of the initial RA treatment strategy that should be gradually reduced and stopped within three months. Thus, no patient was on corticosteroids at the onset of the participant enrollment phase. However, we added prednisolone at the lowest dose and for the shortest possible period during disease flares.

In addition, the eligible participants should have no history of tuberculosis, no active tuberculosis, and no history or evidence of latent tuberculosis. Thus, a negative chest X-ray was required to demonstrate the absence of tuberculosis. Each participant signed written informed consent. The Institutional Review Boards (IRBs) of King Abdulla University Hospital (11/99/2016) and Hamza Hospital (32/2032) approved the conduct of this study. The study was conducted according to the current International Council for Harmonization Good Clinical Practice, the Declaration of Helsinki, and applicable local regulations. The study protocol had been registered on the ClinicalTrials.gov website, with the unique identifier (NCT number) of NCT03348046. This research was funded by Hikma Pharmaceuticals LLC, Amman, Jordan, with the Grant Number of RMS-JOR-2015-01.

### 2.3. Study Outcomes and Assessment Tools

The primary endpoint was the safety of IFX biosimilar by monitoring the adverse events (AEs). The AEs were reported per the guidelines of the International Council for Harmonization Good Clinical Practice, Medical Dictionary for Regulatory Activities (MedDRA/J), and Office for Human Research Protections (OHRP) [14–16]. The AE is identified as any untoward medical event in which the study subject administered a pharmaceutical product, and this event does not necessarily have a causal relationship with the product. Thus, any unfavorable and unintended subjective or objective findings are considered AEs, including symptoms, signs, disease, or laboratory test result changes based on the standard local laboratory reference range, whether the event was related or unrelated to the studied product [14–17].

The incidence of AEs occurrence was assessed and recorded every four weeks throughout the study course up to and including the 34th week. Also, the drug discontinuation due to AEs and the potential causal relationship of AEs to the study drug were evaluated and reported. The causality relationship was assessed based on biological plausibility, prior experience with the drug, and the temporal relationship between drug initiation and AE onset [14–17].

The AEs were further categorized according to their expectedness. Unexpected AE is recognized as the nature or severity of the event being not consistent with the applicable SmPC of IFX biosimilar [14–17]. The determination of AE expectedness, as either an expected or unexpected event, is made by the sponsor on a case-by-case basis and not by the reporter of the AE or the principal investigator [15, 17]. This approach helps ensure compliance and avoid underreporting and allows the sponsor to provide its evaluation in the full context of the drug safety database [17].

In addition, the severity of each AE was investigated. Thus, the AEs were classified into mild, moderate, or severe events [16]. Mild AEs included transient, easily tolerated observations of the minor irritant type that do not interfere with normal daily activities and do not require therapy or medical intervention. Moderate AEs represented those associated with a low level of concern or inconvenience to the participant that may interfere with their daily activities but are improved by a minimal, local, and noninvasive medical intervention. Lastly, medically significant, usually incapacitating events that interrupt the participant's daily activities and require systemic therapy are classified as severe AEs. However, severe AEs are not life-threatening, not hospitalization indicated, and not disabling. Each event that is life-threatening, causes or prolongs inpatient hospitalization, or results in a significant disability or incapacity, a congenital anomaly, a birth defect, or even death, regardless of the drug dosage, is categorized as a serious adverse event (SAE) [14–17]. The SAEs should be reported immediately to the sponsor.

The secondary endpoint was the effectiveness of IFX biosimilar by investigating the mean changes in the 28-Joint Disease Activity Score (DAS28) and the Health Assessment Questionnaire Disability Index (HAQ-DI). Also, the changes in the laboratory values of ESR were recorded. These endpoints were measured at baseline and weeks 14 and 30.

DAS28 is a scoring system widely used to assess RA patients' treatment effectiveness and disease activity in daily practice [18–20]. It is calculated from four parameters: two of them are subjective components, including tender joints (range 0–28) and Patient Global Assessment (range 0–100), while two of the parameters are objective, including swollen joints (range 0–28) and laboratory value of C-reactive protein (CRP) or ESR [18, 21, 22]. However, several studies reported that DAS28 using CRP values underestimates disease activity and overestimates response to treatment [23–28]. In addition, to avoid the discrepancy in DAS28 results in our study and because of the lack of numerical CRP values for two participants, we calculated the DAS28 using the ESR laboratory values (DAS28-ESR) for the enrolled patients. Further, the disease activity was interpreted using the DAS28-ESR validated thresholds into high (>5.1), moderate (3.2–5.1), or low (2.6–3.1) disease activity, while remission was defined as DAS28-ESR <2.6 [21, 27, 29, 30].

HAQ-DI is a functional status assessment tool that evaluates the difficulties patients have had in performing activities of daily living over the past week [31–37]. It comprises 20 items categorized into eight categories of daily function, including dressing and grooming, arising, eating, walking, hygiene, reaching, gripping, and errands and chores. There are 2 or 3 items for each section. For each item, there is a four-point Likert response score of ability, ranging from 0 to 3, with higher scores indicating more functional difficulty (0 = no difficulty; 1 = some difficulty; 2 = much difficulty; and 3 = inability to perform). In addition, each item on the HAQ-DI has a companion aids or devices variable that is used to record the type (s) of needed assistance to achieve the item activity.

Scoring the HAQ-DI is started by giving each category a single score equal to the maximum value (highest score/worst score) reported by the patient for any component item of that category (0, 1, 2, or 3). Then, adjust if aid or device is used or if help is required from another individual by increasing the category score from zero or one to a score of two. If the category score is already ≥2, no adjustment is made. After that, we sum the answered categories' scores and divide the final sum by the number of answered categories to obtain the overall HAQ-DI score. Answering at least six of the eight categories is required to compute the overall HAQ-DI score, or else the HAQ-DI cannot be computed. The overall score of HAQ-DI has a continuous value ranging from zero to three, with 0.125 increments. Thus, the HAQ-DI scale has 25 possible values (i.e., 0, 0.125, 0.250, 0.375,…, 3). The higher the HAQ-DI score, the more functional the disability of the patient: a zero score indicates no functional impairment, scores of 0.125–1 indicate mild to moderate disability, and scores of 1.125–2 represent moderate to severe disability, while scores of 2.125–3 indicate severe to a total disability [34–37].

### 2.4. Statistical Analysis

Safety data were summarized descriptively, including AEs, SAEs, and medication discontinuation due to AEs. The mean (SD) values of ESR, DAS28-ESR, and HAQ-DI scores at the baseline, 14th week, and 30th week were compared and tested for statistical significance using paired *t*-test. The alpha level was set at a *p* value of 0.05 for statistical significance. The *t*-test critical values, degree of freedom (d*f*), *p* values, and 95% confidence intervals (95% CI) were reported for the paired *t*-test differences in the mean scores of DAS28-ESR and HAQ-DI scales over the study. Further, Cohen's *d* statistics was conducted to measure the effect size for the paired *t*-test changes. Thus, the score change was categorized based on the effect size into no effect (<0.20), small effect (0.20–0.49), moderate effect (0.50–0.79), and large effect (≥0.80) [38–40].

## 3. Results

### 3.1. Disposition and Demographics

**Figure 1:**
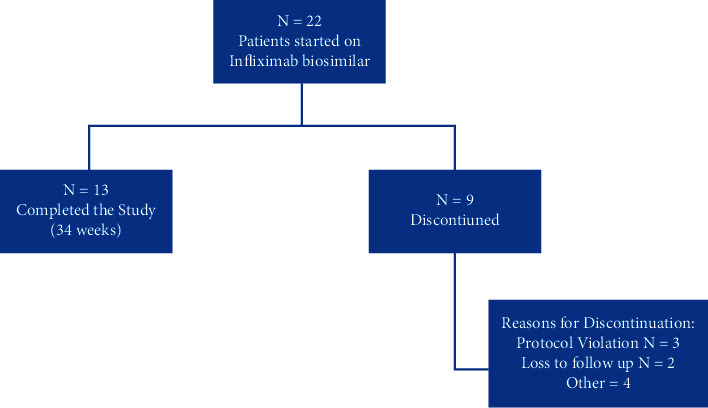
Flow diagram of rheumatoid arthritis patients who started Infliximab biosimilar.

A total of 22 patients agreed to participate in this study and initiated IFX biosimilar, of which 12 (54.5%) patients were enrolled from KAUH and ten (45.5%) from PHH (Figure 1). A total of 13 participants completed the 11 clinic visits after IFX biosimilar initiation, while nine (41.0%) patients discontinued the study. More specifically, three patients were discontinued due to protocol deviations, including noncompliance with IFX biosimilar by two patients, and the third one missed study visits per study protocol due to medical insurance issues. In addition, two patients were lost to follow-up, and four were discontinued for other reasons. However, no patients discontinued the study due to AEs, and the dropped-out patients' data were analyzed up to the point of withdrawal from the study.

The mean (SD) age of the enrolled patients was 44.64 (10.40) years, and around two-thirds were female participants. The mean (SD) duration of the disease was 6.19 (5.61) years, and the vast majority of enrolled patients (86.4%) were on oral methotrexate throughout the study period with no dosage changes. The demographics and baseline characteristics of enrolled patients were similar between the two study sites (Table 1). The mean (SD) of the DAS28-ESR score at the baseline was 6.52 (0.23), representing severe disease activity, and no significant differences in DAS28-ESR scores were observed between both sites. Differences in HAQ-DI scores, CRP, and ESR levels were noted between the patients of the two sites.

The total number of administered IFX biosimilar doses during this study was 101, representing 76.5% of the total 132 planned doses for the 22 enrolled patients. As mentioned earlier, this dropout in doses was attributed to the loss of follow-up of nine patients during the study conduction because of protocol deviations or other reasons. The mean (SD) IFX biosimilar dose consumed at the baseline visit was 269.23 (85.48) mg for the 22 enrolled patients, and it slightly decreased to 263.63 (58.10) mg for the 13 patients at the last dose of the 11th visit. The median number of doses was five, and the median duration of exposure was 226.5 days per patient throughout the study.

### 3.2. Safety as a Primary Endpoint

A total of 35 AEs were reported in 14 patients out of the enrolled participants (63.6%). Among them, two (5.7%) events were SAEs and reported in two patients. One patient experienced typical cardiac chest pain at week 33 of the study, and the symptom faded spontaneously with normal echocardiogram and catheterization results. Thus, this SAE was reported as a spontaneously recovered symptom. The other patient experienced lower limb edema at week 14 that was not recovered until the end of the study. The latter patient received Furosemide as a concomitant medication in week 14 after the SAE started. Both SAEs were unrelated to the study medication. The principal investigator initially stated these two SAEs to the sponsor as unexpected SAEs. After that, the sponsor revised them, which were found to be listed in the SmPC; thus, they were reported as expected SAEs. Nevertheless, out of the 35 AEs, 12 (34.3%) unexpected AEs were reported; none were severe or related to the study medication.

Besides the previously reported two SAEs, 31 (88.6%) AEs were either mild (*n* = 5, 14.3%) or moderate (*n* = 26, 74.3%), while two events (5.7%) were severe. Most AEs (*n* = 28, 80.0%) were recovered spontaneously. Three AEs (8.6%) were reported as recovering with medications, and four AEs (11.4%) were not recovered. Two AEs in two patients were related to the study medication, including leukopenia and herpes zoster; however, none were serious. None of the subjects permanently discontinued treatment or withdrew from the study due to AEs, and no deaths were reported.

**Figure 2:**
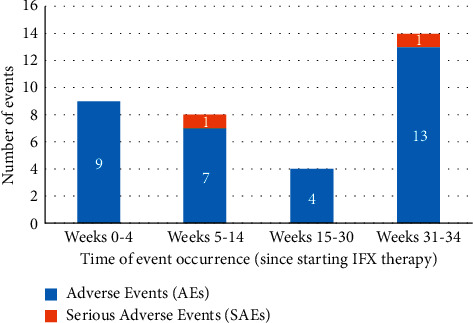
Timeline of adverse events (AEs) and serious adverse events (SAEs) during the study.

The most frequently reported AEs among participants were productive cough (13.6% of patients), a decrease in hemoglobin (13.6% of patients), and pyrexia (13.6% of patients). Chest pain and arthralgia were reported in 9.1% of patients for each (Table 2). The reported 35 AEs corresponded to 0.61 events per 100 patient-days (PDs), with the highest frequency for pyrexia (14.17 events per 100 PDs) and arthralgia (13.81 per 100 PDs) (Table 3). Adverse events were reported at different times during the study (Figure 2). Most of the AEs were shown to occur between week 31 and the end of the study.

### 3.3. Effectiveness as a Secondary Endpoint

The IFX biosimilar effectiveness of participants' data is presented for the whole analysis sample (*n* = 22) utilizing DAS28-ESR, HAQ-DI, and ESR values.

The mean (SD) scores of DAS28-ESR decreased significantly from 6.55 (1.16) at baseline, representing a severe disease activity, to 4.59 (1.45) at week 14 (t (d*f*, 21) = −7.93, *p* < 0.0001, 95% CI −2.474 −( −1.445)) with a large effect of difference (Cohen's *d* = 1.69). At week 30, the mean (SD) DAS28-ESR score became 4.77 (1.09) (*t* (21) = −7.20, *p* < 0.0001, 95% CI −2.294 −( −1.266)), with a large effect of difference (Cohen's *d* = 1.53). At weeks 14 and 30 of the study, the disease activity was moderate based on DAS28-ESR mean scores.

**Figure 3:**
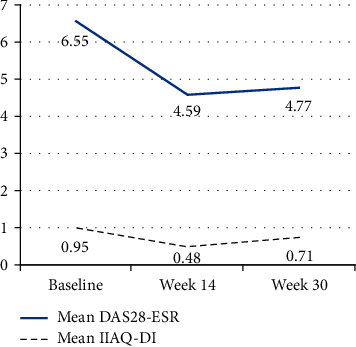
Mean scores of the 28-Joint Disease Activity Score using Erythrocyte Sedimentation Rate (DAS28-ESR) and disability index of the health assessment questionnaire (HAQ-DI) at baseline, week 14, and week 30.

Regarding HAQ-DI, the mean (SD) scores significantly decreased from 0.95 (0.74) at baseline to 0.48 (0.62) at week 14 (*t* (21) = −2.98, *p*=0.008, 95% CI −0.798 −( −0.142)). This change was of moderate effect, with Cohen's *d* = 0.64. At the end of the study, week 30, the mean (SD) HAQ-DI score returned back to 0.71 (0.78), with no statistically significant difference as compared to the baseline score (*t* (21) = −1.52, *p*=0.483, 95% CI −0.568–0.088). However, this difference was of small effect (Cohen's *d* = 0.32) (Figure 3).

**Figure 4:**
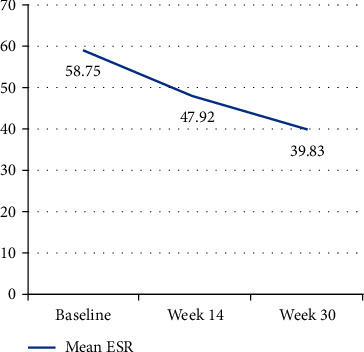
Mean Erythrocyte Sedimentation Rate (ESR) values at the baseline and weeks 14 and 30.

The mean (SD) value of ESR decreased from 58.75 (26.94) at baseline to 47.92 (33.89) at week 14 (*p*=0.082) and to 39.83 (17.38) at week 30 (*p*=0.005) (Figure 4).

## 4. Discussion

In real-life clinical settings, this postauthorization study highlighted the safety and effectiveness of IFX biosimilar as part of the IFX biosimilar Risk Management Plan (RMP) for JFDA. Most reported AEs were mild or moderate, and no AEs led to the discontinuation of the study medication. The reported AEs align with the known safety profile of the standard IFX original [3–5]. The high effectiveness of IFX biosimilar in managing RA was approved in this study, with observed significant improvements in disease activity, functional ability, and inflammatory markers throughout the follow-up period. These improvements achieved over 34 weeks would be reflected in better long-term outcomes, as some or all of the improvements will last beyond the study. Thus, despite the higher price of IFX biosimilar compared to the currently available therapies, the cost-effectiveness of IFX biosimilar treatment should be considered in light of the long-term benefits and maintaining the patient's ability to work.

The mean DAS28-ESR scores significantly decreased from baseline to week 14 and from baseline to the end of the study. The mean HAQ-DI scores significantly decreased from baseline to week 14, but the reported decrease in HAQ-DI scores between baseline and the end of treatment was statistically nonsignificant (*p* > 0.05). This finding could be attributed to the high health risks in our cohort, such as the high body mass index (BMI), long disease duration, and high baseline DAS28 score in our patients. These factors were recognized as negative predictors for improvement in HAQ-DI scores over six months [41]. In addition, we could not exclude the floor effect in which patients with low baseline HAQ-DI scores cannot achieve significant decreases in HAQ-DI despite observed clinical improvements [35, 41]. In our cohort, the baseline score of the HAQ-DI was low, with a mean value of 0.95. Behrens et al. reported the baseline HAQ-DI score as a significant predictor for improvement in HAQ-DI and indicated that the critical difference for change beyond random variation in the HAQ-DI was low (0.597) for patients with baseline HAQ-DI < 1, while it was high (0.673) for patients with baseline HAQ-DI ≥ 1. In our study, the enrollment criteria included RA patients regardless of their disease activity and functional status at baseline; thus, those who were with good physical function (HAQ-DI ≤ 0.5) or had low disease activity (DAS28 ≤ 3.2) at baseline were included in the analyses. These patients are not well suited for this measure because of the significant change required to achieve a HAQ-DI score improvement response. Also, several studies concurred on the impact of pain on functional status and identified the baseline and change in pain over time as the most significant component of HAQ-DI and the essential negative predictor for HAQ-DI improvement [41–44].

Rather than clinical issues, lower HAQ-DI improvements at the end of the study could be attributed to statistical issues, such as the observational design of the study, the small sample size, the short observation period, and the random fluctuations in the measured scores. In addition, it is critical to note that patients may experience meaningful benefits with HAQ-DI improvements lower than the statistically significant HAQ-DI changes. However, despite the lack of statistical significance in HAQ-DI difference between the onset and end of the study, this difference has a small effect, with Cohen's *d* = 0.32, which is higher than the predetermined minimum clinically important difference (MCID) of 0.22 for the HAQ-DI [41, 45–47]. In addition, the reported mean HAQ-DI scores in our study represented mild to moderate difficulty with activities of daily living throughout the study duration, and the reported improvements with the use of IFX biosimilars in this study are concordant with the findings of the previous clinical trials and observational studies [2, 4].

At the beginning of the study, we planned to enroll 40 patients with active RA who are biologically naïve attending the study sites with an expected recruitment time of 12 months and a monitoring treatment period of 34 weeks, with 11 clinic visits for each patient. However, the patients' recruitment process was slow and challenging. It started at the KAUH site in Irbid city in January 2017, but it collides with limited financial resources and the unavailability of health insurance for some patients. Thus, only a few patients were recruited from KAUH.

At the PHH site, a Ministry of Health hospital in Amman city, the first patient was recruited in October 2017, a ten-month delay after starting the patient recruitment process at the KAUH site. This delay in the recruitment process was attributed to the prolonged period needed to get the IRB approval from the Ministry of Health and hospital administration, as the approval was received in August 2017. Moreover, the patient recruitment process in the PHH was affected by the unavailability of IFX biosimilar in that hospital. IFX biosimilar was out of stock in PHH for three months, including December 2017, March 2018, and April 2018, which caused a delay in the enrollment of more patients from the PHH site in this study. Thus, the recruitment process was put on hold during these times due to IFX biosimilar unavailability, and ultimately the study was stopped for the same reason a year later. Nonetheless, the treatment phase of the study was accomplished without medication interpretation until the registered follow-up period of enrolled patients from the PHH site was over, and no patients were off the IFX biosimilar therapy during the study, as the required doses had been kept in the registered stock for every enrolled patient since they enrolled in the treatment phase of the study.

Our findings suggest that managing RA with IFX biosimilar in routine clinical practice improves disease activity and patients' clinical response to the therapy, and this therapy delays the functional impairment of the patients. Thus, the patient's ability to work is expected to be improvedand, and their resource consumption would be lower, consequently enhancing their quality of life (QoL). Previous studies indicated that RA patients' resource consumption increases, and their QoL decreases as the disease progresses [48–50]. As a result, the improvement in disease progression with IFX biosimilar could be associated with potentially considerable cost savings, especially with the potential of the biological drug discontinuation or dose reduction with a successful recovery achievement if the therapy was administered early [51, 52]. Consequently, a better cost-effectiveness ratio could be achieved with IFX biosimilar rather than the currently available therapies, or at least the cost would remain within the usual budget for a therapy to be endorsed, despite the high IFX biosimilar direct costs. This suggestion is concordant with Jha et al. reporting the one-year cost savings ranging between €25.79 and €77.37 million (10%–30% discount) with the introduction of IFX biosimilar (Remsima®) for the management of six inflammatory autoimmune diseases, including RA, across five European countries as compared with the standard IFX original (Remicade®) [53].

### 4.1. Strengths and Limitations of the Study

The novelty of this study is in providing the first real-life evidence from a developing country's clinical settings on the safety and effectiveness of IFX biosimilar among RA patients and in its prospective multistage format with up to 11 clinic visits for each patient over a follow-up monitoring period of 34 weeks of IFX biosimilar administration. Also, the positive data about the IFX biosimilar provided by this study would further encourage its use in rheumatology in developing countries, thus, providing clinical benefits to the patients and achieving cost savings for the healthcare system.

Still, several issues should be reported as limitations in interpreting the study results. First, this study was observational, with no control group treated with the standard IFX original to compare with. Thus, we could not provide evidence for causal relationships, the selection bias could not be ruled out, and the lack of statistical significance may be attributed to the lack of power to detect differences under observation. Second, the sample size was relatively small, and the dropout rate was considerably high. However, the sample size of 22 patients with 14 patients (63.6%) developed AEs as the primary outcome of this study has a statistical power of 78.2% with a 0.05 alpha level in light of previous studies' findings [54]. Also, the sample size was primarily based on the available pool of patients eligible for biological therapy as decided by the official committee from the two registered hospitals. This further contributes to limiting the sample size, introducing a selection bias, and causing dropouts. Due to the high reported dropout rate in this study, we did not exclude the data of the discontinued patients. Further, their data was analyzed up to the point of withdrawal from the study.

Third, the DAS28-ESR and HAQ-DI measures' data were unavailable in all study visits due to the shift of visit times for many patients. It is important to note that this study was a real-life multicenter observational cohort study in a developing county, Jordan, and thereby, the collected data would reflect the defects in medical practice and management of RA in Jordan. Thus, high dropout, shifting of clinic visits, unavailability of medication, and missing data are significant factors in reducing the number of enrolled participants, which is standard practice in such scenarios. Finally, the availability of medications within the recruitment sites follows bureaucratic governmental protocols that delay drug availability. Thus, the small sample size and the deviation from the recruitment plan affected our results and may have diluted the observed differences.

## 5. Conclusions

This multicenter prospective study supports the IFX biosimilars as a well-tolerated, safe, and effective therapy in managing RA patients by applying naturalistic clinical settings in Jordan. Highly significant and comparable improvements were detected in disease activity, functional ability, and inflammatory markers, with no significant AEs. Thus, the cost-effectiveness of IFX biosimilars should be considered in light of the long-term benefits rather than the direct drug costs, as most of the savings will come from maintaining the functional ability of the RA patients. This is particularly important when considering new expensive therapies in chronic progressive inflammatory diseases. A larger sample size of rheumatologic patients and a more extended follow-up period are recommended in any future study. However, we still believe that the findings of this study qualify as novel and valid as they come from a developing country with limited health resources, and the reported safety and effectiveness over 34 weeks of follow-up will last beyond the study period. Also, the close alignment of our findings with other studies strengthens the weight of evidence that IFX biosimilar is a safe, effective, and cost-effective alternative to the standard IFX original. Thus, this study provides a real-world landmark for utilizing the new generations of biosimilars for treating chronic inflammatory conditions in developing countries.

## Figures and Tables

**Table 1:** Demographics and baseline characteristics of participants and their differences by the study site.

Variable	Total cohort, *n* = 22	Site 1-KAUH, *n* = 12 (54.5%)	Site 2-PHH, *n* = 10 (45.5%)	*p* value
Female, *n* (%)	16 (72.7)	9 (75.0)	7 (70.0)	0.793

Age in years, mean (SD)	44.64 (10.40)	42.50 (11.21)	47.21 (9.42)	0.284

Height in cm	*n* = 15	*n* = 12	*n* = 3	0.344
Mean (SD)	165.9 (9.2)	165.1 (9.9)	169.3 (5.9)	

Weight in kg	*n* = 17	*n* = 12	*n* = 5	0.512
Mean (SD)	82.7 (15.5)	81.3 (17.1)	86.0 (11.6)	

BMI	*n* = 15	*n* = 12	*n* = 3	0.419
Mean (SD)	29.4 (5.9)	29.9 (6.3)	27.6 (3.8)	

RA duration in years, mean (SD)	6.19 (5.61)	6.66 (6.10)	5.56 (5.11)	0.645
Min	0	0	2	
Max	19.0	19.0	18.0	

RF positive, *n* (%)	22 (100.0)	12 (100.0)	10 (100.0)	—

Concomitant medications:				
Methotrexate use, *n* (%)	19 (86.4)	10 (83.3)	9 (90.0)	0.650
NSAID use, *n* (%)	8 (36.4)	1 (8.3)	7 (70.0)	0.002

DAS28-ESR	*n* = 20	*n* = 12	*n* = 8	0.603
Mean (SD)	6.52 (0.23)	6.41 (0.89)	6.68 (1.28)	
Min	4.20	4.59	4.20	
Max	7.90	7.60	7.90	

HAQ-DI	*n* = 20	*n* = 12	*n* = 8	0.191
Mean (SD)	0.99 (0.81)	1.18 (0.75)	0.71 (0.81)	
Min	0	0	0	
Max	2.55	2.40	2.55	

ESR, mm/hour	*n* = 21	*n* = 11	*n* = 10	0.059
Mean (SD)	58.57 (26.27)	48.64 (17.55)	69.50 (30.63)	
Min	17.00	27.00	17.00	
Max	128.00	80.00	128.00	

CRP^a^, mg/l	*n* = 19	*n* = 9	*n* = 10	0.576
Mean (SD)	39.55 (85.72)	52.00 (124.82)	28.34 (23.55)	
Min	0	0	5.20	
Max	384.00	384.00	76.60	

^a^Three CRP values for two patients were replaced by “0.0” as the principal investigator filled their original values as “Negative.” KAUH: King Abdullah University Hospital; PHH: Prince Hamzah Hospital; BMI: body mass index; RA: rheumatoid arthritis; RF: rheumatoid factor; DAS28: 28-Joint Disease Activity Score; ESR: Erythrocyte Sedimentation Rate; HAQ-DI: Disability Index of the Health Assessment Questionnaire; CRP: C-reactive protein.

**Table 2:** Adverse effects of Infliximab biosimilar in the safety population and their outcomes.

Adverse event	No. of patients, *n* (%)^a^	Time of occurrence (weeks since starting IFX therapy)	No. of events	Outcome
Blood and lymphatic system disorder				
Anemia	1 (4.5)	12	1	Not recovered
Increased blood pressure	1 (4.5)	34	1	Recovered
Increased blood urea	1 (4.5)	31	1	Recovered
Leukopenia	1 (4.5)	7	1	Recovered

Cardiac disorders				
Chest pain	2 (9.1)	33, 34	2	Recovered
Dyspnea	1 (4.5)	7, 32	2	Recovered

Gastrointestinal disorder				
Gastrointestinal disorder	1 (4.5)	12	1	Recovered
Oropharyngeal pain	1 (4.5)	2	1	Recovered

General disorders and administration site conditions				
Pyrexia	3 (13.6)	2, 4, 32	3	Recovered

Infection and infestation				
Bronchitis	1 (4.5)	24	1	Recovered
Herpes zoster	1 (4.5)	1	1	Recovered

Investigation				
Decreased blood creatinine	1 (4.5)	27	1	Recovered
Increased blood creatinine	1 (4.5)	31	1	Recovered
Decreased hemoglobin without anemia	3 (13.6)	23, 32, 32, 34	4	Recovering/recovered

Nervous system disorder				
Dizziness	1 (4.5)	8	1	Recovered

Metabolism and nutrition disorders				
Peripheral edema	1 (4.5)	14	1	Not recovered

Musculoskeletal and connective tissue disorder				
Arthralgia	2 (9.1)	2	2	Recovered

Renal and urinary disorder				
Dysuria	1 (4.5)	14	1	Not recovered
Renal colic	1 (4.5)	34	1	Recovered

Respiratory, thoracic, and mediastinal disorders				
Productive cough	3 (13.6)	2, 3, 16, 31	4	Recovered
Rhinorrhea	1 (4.5)	2	1	Recovered
Sputum discolored	1 (4.5)	33	1	Recovered
Wheezing	1 (4.5)	33	1	Recovered

Skin and subcutaneous tissue disorders				
Alopecia	1 (4.5)	8	1	Not recovered

Total	**14 (63.6)**	**0–34**	**35**	—

^a^Percentage represents the frequency of patients who developed the adverse event and was calculated by dividing the number of participants who developed the adverse event by the total sample size (*n* = 22).

**Table 3:** Adverse events per 100 patient-days.

Preferred term^a^	Percent of patients with adverse events^b^	No. of events	Adverse events per 100 patient-days
Blood and lymphatic system disorder			
Anemia	4.5	1	1.15
Increased blood pressure	4.5	1	0.22
Increased blood urea	4.5	1	0.46
Leukopenia	4.5	1	2.00

Cardiac disorders			
Chest pain	9.1	2	0.81
Dyspnea	4.5	2	2.34

Gastrointestinal disorder			
Gastrointestinal disorder	4.5	1	1.19
Oropharyngeal pain	4.5	1	6.67

General disorders and administration site conditions			
Pyrexia	13.6	3	14.17

Infection and infestation			
Bronchitis	4.5	1	0.60
Herpes zoster	4.5	1	12.50

Investigations			
Decreased blood creatinine	4.5	1	0.25
Increased blood creatinine	4.5	1	0.46
Decreased hemoglobin	13.6	4	1.9

Nervous system disorder			
Dizziness	4.5	1	1.79

Metabolism and nutrition disorders			
Edema peripheral	4.5	1	0.99

Musculoskeletal and connective tissue disorder			
Arthralgia	9.1	2	13.81

Renal and urinary disorder			
Dysuria	4.5	1	1.01
Renal colic	4.5	1	0.24

Respiratory, thoracic, and mediastinal disorders			
Productive cough	13.6	4	7.61
Rhinorrhea	4.5	1	6.67
Sputum discolored	4.5	1	0.40
Wheezing	4.5	1	0.40

Skin and subcutaneous tissue disorders			
Alopecia	4.5	1	1.79

Total	**63.6**	**35**	**0.61**

^a^Medical Dictionary for Regulatory Activities, version 22.1. ^b^Patients experiencing the same adverse event multiple times were counted every time for the corresponding preferred term.

## Data Availability

The data used to support the findings of this study are available from the corresponding authors upon request.
